# Implementing portable, real-time 16S rRNA sequencing in the healthcare sector enhances antimicrobial stewardship

**DOI:** 10.1016/j.ebiom.2026.106317

**Published:** 2026-06-10

**Authors:** Edward Cunningham-Oakes, David Carlisle, June Booth, Andrew Frankland, Michael McDowell, Jack Pilgrim, Aleksandra Rzeszutek, Ceri Evans, Susan Larkin, Ang Li, Christopher Loftus, Merna Samuel, Nicola Scott, Luke Swithenbank, Victoria Owen, Alistair C. Darby, Anna Smielewska

**Affiliations:** a Department of Infection Biology and Microbiomes, Institute of Infection, Veterinary and Ecological Sciences, University of Liverpool, Liverpool, United Kingdom; b NIHR Health Protection Research Unit in Gastrointestinal Infections, Liverpool, United Kingdom; c Liverpool Clinical Laboratories, CSSB, Liverpool, United Kingdom; d Department of Evolution, Ecology and Behaviour, Institute of Infection, Veterinary and Ecological Sciences, University of Liverpool, Liverpool, United Kingdom; e School of Food Science and Nutrition, University of Leeds, Leeds, United Kingdom; f Alder Hey Children’s Hospital, Liverpool, United Kingdom; g Royal Liverpool University Hospital, Liverpool, United Kingdom

**Keywords:** Antimicrobial, Resistance, 16S, Nanopore, Sequencing, Diagnostics

## Abstract

**Background:**

Antimicrobial resistance (AMR) poses a significant global health challenge, resulting in over 1.27 million deaths in 2019 and is projected to cause up to 10 million deaths annually in the future. To address this issue, the healthcare sector requires rapid (24–72 h) and accurate (to genus or species-level) bacterial identification. We implemented 16S ribosomal RNA (rRNA) sequencing using Oxford Nanopore Technology (ONT) in an NHS setting to enhance diagnostic capabilities, reduce antibiotic misuse and improve patient outcomes.

**Methods:**

We used ONT-based 16S rRNA sequencing on sterile site samples (pus, fluid, tissue) from seven NHS hospitals in Cheshire and Merseyside, England. The assay, validated against Sanger sequencing and MALDI-TOF, had a 24–72 h turnaround. Clinical impact was assessed by tracking antibiotic changes and patient outcomes over several months.

**Findings:**

ONT 16S sequencing informed antimicrobial stewardship in 56.9% of cases (124/218). It was mainly used when cultures failed (32.1%) or when patients were already on antibiotics (32.6%). Results often confirmed existing therapy (26.6%) or led to no change (28%) but still supported targeted prescribing. *Streptococcus* and *Staphylococcus* were most frequently detected genera, with *Streptococcus* common in ICU samples. The assay had the highest clinical impact in patients who are immunosuppressed, improving treatment precision.

**Interpretation:**

The integration of ONT 16S sequencing into routine NHS diagnostics has enabled antimicrobial stewardship by offering a faster method with improved taxonomic resolution. Its earlier use in cases where routine cultures are likely to fail may contribute additional microbiological information for antimicrobial decision-making and may reduce diagnostic uncertainty.

**Funding:**

Liverpool Clinical Laboratories (LCL) funded this work.

**Research in context**

**Evidence before this study**

We conducted a comprehensive literature review to assess the current evidence on the clinical use of 16S ribosomal RNA (rRNA) sequencing with Oxford Nanopore Technology (ONT) for bacterial identification and its potential impact on antimicrobial stewardship. Using PubMed, we searched for studies published up to December 31, 2024, employing terms such as “Oxford Nanopore,” “16S rRNA sequencing,” “clinical implementation,” “bacterial identification,” and “antimicrobial stewardship.” We included studies that explored the use of ONT 16S rRNA sequencing in clinical environments, focusing on diagnostic accuracy, turnaround time, and implications for antimicrobial prescribing. The search revealed a limited number of studies examining the routine clinical application of this technology within the NHS, with few addressing its direct influence on prescribing practices.

**Added value of this study**

This study represents a pioneering effort in the UK, integrating ONT 16S rRNA sequencing as a routine diagnostic tool across multiple NHS hospitals. The approach enables the rapid identification of bacterial pathogens, with urgent cases yielding results within 24 h. In our cohort, 124 out of 218 (56.9%) 16S samples directly contributed to antimicrobial stewardship, either by rationalising (103 cases) or escalating (21 cases) antibiotic therapy. The greatest impact was observed in patients who are immunocompromised, who had significantly higher odds of a stewardship-driven intervention (OR = 2.99, 95% CI [1.11–9.50], p = 0.04) compared to patients who are immunocompetent. Age and sex were not significantly associated with stewardship impact. Across all patient groups, 16S sequencing reduced diagnostic uncertainty and supported more targeted antimicrobial use, demonstrating a measurable clinical benefit.

**Implications of all the available evidence**

The routine integration of ONT 16S rRNA sequencing into clinical workflows offers a transformative pathway to improving diagnostic precision and speed in bacterial infections. Our findings indicate that this approach can meaningfully influence antimicrobial stewardship, particularly for patients who are immunocompromised, by supporting both escalation and rationalisation of therapy. Future research should aim to evaluate the performance of this technology across diverse healthcare settings and its long-term impact on patient outcomes and resistance patterns.

## Introduction

Antimicrobial resistance (AMR) is one of the most critical health challenges of the 21st century.^1^ This silent pandemic, facilitated by a stagnant antibiotic pipeline and the need for improved antimicrobial stewardship,^2^ is already responsible for significant illness and death globally. Recent data indicate that in 2019 alone, 1.27 million deaths were linked to AMR, and this number is projected to reach up to ten million in the coming years.^3^ AMR is also listed as one of the four chronic risks on the UK National Risk Register along with Climate Change, Risks posed by AI and Organised Crime.^4^

The same document recognises the key importance of both the reduction as well as the optimisation of antimicrobial use. Rapid and precise diagnostics are the first step towards these goals.^5^ However, the current methods in routine diagnostic laboratories do not always facilitate this need. Traditional culture methods are unsuitable for fastidious or slow-growing organisms^6^ and samples taken post-antibiotic therapy, whilst molecular methods are restricted to a specific set of organisms.^7^ Real-time, accredited clinical metagenomic assays that can be deployed locally and show real clinical impact are more important than ever,^8^ given the increasing threat posed by antimicrobial resistance.

The 16S ribosomal RNA (rRNA) gene is ubiquitous in prokaryotes.^9^ Direct and rapid diagnostics from clinical samples using 16S sequencing (metataxanomics) falls broadly under the umbrella of clinical metagenomics.^8^ This method of rapid pathogen identification offers advantages for preventing antimicrobial resistance by enabling rapid pathogen identification. This allows tailoring of treatment and facilitates antimicrobial stewardship. Moreover, this approach is not limited by bacterial culture^10^ and is suitable for detecting complex polymicrobial infections, including rare organisms in patients experiencing polypharmacy.^10^ Simultaneously, new NHS assays would benefit from lean bacterial diagnosis, which refers to a streamlined diagnostic process aligned with lean thinking in healthcare, aiming to improve patient outcomes and resource efficiency by reducing delays, length of stay, and unnecessary steps.^11^

Here, we outline the implementation of whole gene 16S sequencing using Oxford Nanopore Technology with fast turnaround time (24–72 h) and lean bacterial diagnosis and identification for improved antimicrobial stewardship. We describe the clinical impact of utilising this technology on antimicrobial prescription decisions across seven different hospitals and provide extensive benchmarking against externally accredited standards. This work will serve as a guide to those seeking to implement pathogen diagnostics as clinically-accredited assays, thereby bridging the gap between genomics and clinical diagnostics.

## Methods

### Ethical approval

This quality improvement project utilised surplus diagnostic materials and reference material provided for this purpose to improve service quality and as part of the diagnostic laboratory quality assessment. It is not classified as research under the UK Clinical Research Collaboration. Therefore, it does not require ethical approval. However, the work was approved by Liverpool Clinical Laboratories (LCL), Liverpool University Hospitals NHS Foundation Trust, and NHS England under the UK Health Security Agency's UK Standards for Microbiology Investigations (UK SMI) Evaluations, Verifications, and Validations of Diagnostic tests.

### Sample collection for assay validation

Prior to the introduction of the test, a total of 109 samples were included in the assay validation part of the project. Of these, 104 were surplus clinical samples and five were from an external quality control scheme; Quality Control for Molecular Diagnostics (QCMD).^12^ All clinical samples used were surplus material that had been stored at −20 °C by the routine diagnostic microbiology laboratories involved in the project. Samples were included if they had sufficient residual volume for testing and met assay quality control thresholds, including successful detection of all eight target organisms to at least genus level during sequencing and a post-cleanup Qubit concentration >5 ng/μL after PCR. No additional exclusion criteria were applied.

### Extraction from samples

For tissue samples, a petri dish, forceps, and scalpel were used to cut a small sample piece, which was transferred to a UV-irradiated MagNA Lyser green bead tube (Roche Diagnostics; Cat# 03358941001) containing 400 μL Roche Bacterial Lysis Buffer (BLB). The tube was heated at 95 °C for 10 min, then allowed to cool before placement in the MagNA Lyser for 50 s at a setting of 6500 rpm. The sample was then centrifuged for 10 min at 8000 rpm, and 400 μL was transferred into a new UV-irradiated 2 mL tube containing 25 μL protease and 6 μL carrier RNA.

For fluid samples, 200 μL of sample was added to a tube containing 200 μL UV-irradiated BLB, 25 μL protease, and 6 μL carrier RNA. The mixture was vortexed for 10 s, centrifuged for 10 s at 8000 rpm, and heated at 95 °C for 10 min, then allowed to cool before being transferred to the Qiagen Qiacube instrument, where on-screen instructions were followed.

A positive and negative Internal Quality Control (IQC) were extracted in parallel with each clinical sample. The positive IQC, ZymoBIOMICS Microbial Community Standard II, (Zymo Research; Cat #D6310), was stored at −80 °C and vortexed vigorously before use, with 75 μL used per extraction. The negative control consisted of UV-irradiated BLB only. For positive control extraction, 75 μL control material was added to a tube containing 325 μL UV-irradiated BLB, 25 μL protease, and 6 μL carrier RNA. The mixture was vortexed for 10 s, centrifuged for 10 s at 8000 rpm, heated at 95 °C for 10 min, and allowed to cool before transfer to the Qiacube.

Upon extraction completion, all elutions were quantified using the Qubit Flex Fluorometer and the Qubit dsDNA HS Assay Kit (ThermoFisher Scientific; Cat #Q32851). All elutions were normalised to 1 ng/μL before proceeding.

### Cross-comparison of sequencing to gold-standard clinical results

Our workflow underwent External Quality Assessment (EQA) with QCMD. QCMD provided a series of bacterial samples from three different distribution cycles (2016, 2017 and 2021), for which the correct bacterial identification was known. DNA was extracted from these samples, alongside all clinical samples (described in Table 1) and assessed using a previously accredited method for lean bacterial diagnosis (Sanger sequencing). 27 F and 797 R primers were used to amplify a ∼770 bp region of the 16S gene. Amplicon integrity and size was then confirmed via 1% agarose gel electrophoresis. Products were subsequently purified with ExoSAP-IT™ (ThermoFisher Scientific, Cat #78205.10. ML) by adding 5 μL of the enzyme to the PCR product, followed by incubation at 37 °C for 15 min and 80 °C for another 15 min.

**Table 1:** Summary of clinical sample types submitted for 16S sequencing.

Sample type	Total	Percentage
CSF	52	23.9
Fluid (Other)	41	18.8
Joint Fluid	39	17.9
Tissue	36	16.5
Pus/Abscess	20	9.2
Blood Culture	15	6.9
Swab	7	3.2
Other	4	1.8
Unknown	2	0.9
Line-related	1	0.5
Respiratory	1	0.5

Subsequent PCR was conducted using 1 μL of the purified product with the BigDye™ Terminator v1.1 Cycle Sequencing Kit (ThermoFisher Scientific; Cat #4336697) to incorporate fluorescently tagged bases.^12^ Post-PCR, amplicons were purified again using a two-step ethanol precipitation process: first with 24 μL absolute ethanol and 1 μL 3 M NaAc, followed by centrifugation at 2000 × *g* for 20 min, and then with 75 μL 70% ethanol and centrifugation at 2000 × *g* for 5 min. The precipitated DNA was resuspended in 10 μL Hi-Di Formamide (ThermoFisher Scientific; Cat #4311320) and sequenced on an ABI 3500 genetic analyser (ThermoFisher Scientific; Cat #A3564).

Consensus sequences were generated using SeqScape™ Software and submitted to BLAST (NCBI, Bethesda, MD, USA; RRID:SCR_004870) for organism identification at the genus or species level, following the Clinical & Laboratory Standards Institute (CLSI) guidelines. Results were stored for later comparison. If sequencing did not yield a result, where possible, samples were cultured on Columbia blood agar (Merck; Cat #1465590020) and identified using the Microflex® LRF MALDI-TOF benchtop analyser (Bruker; Cat #43080.2 B). Results for 2023 from both ONT and Sanger sequencing were then reported to QCMD.

### Read-based 16S microbiome profiling and consensus 16S pathogen sequence generation

We developed a simple bioinformatic workflow for analysis of 16S data, both on a read-by-read basis (for both low, and high-abundance samples), and consensus fasta files (high-abundance samples). The workflow was developed to account for the inherent variability in the bioburden of clinical samples, while also taking into account varying computational infrastructures across different Trusts and limited bioinformatics training.

Additional confidence in the validity of results for low-abundance samples was provided by clinical interpretation of results in relation to patient outcome, and additional diagnostic data where available.

Sequencing of libraries generated using a 16S Barcoding Kit (BC1-14) SQK-16S024 (Oxford Nanopore Technologies; RRID:SCR_003756) was performed using R9.4.1 flow cells (FLO-MIN106; Oxford Nanopore Technologies; RRID:SCR_003756). On average, 2 h of sequencing generated sufficient reads for accurate diagnosis. Nanopore sequencing data (reads) were obtained from the experiment in FASTQ format. NanoFilt (v2.8.0; RRID:SCR_016966)^13^ was then used to quality-filter reads based on the specified quality thresholds (Q12, minimum 850 bp, maximum 1500 bp). This threshold was selected based on the recommended input for consensus fasta generation using amplicon_sorter.py (version = ‘2023-06-19').^14^ Reads not meeting the quality threshold were excluded from further analysis, and the remaining reads were merged.

Kraken2 (v2.0.7-beta; RRID:SCR_005484)^15^ was then used to classify the selected Nanopore reads taxonomically, using Greengenes Database (Release 13_5; RRID:SCR_002830), with a confidence threshold of 0.1. These results were visualised in the Pavian^16^ web browser (https://fbreitwieser.shinyapps.io/pavian/; RRID:SCR_016679), to enable accessible interpretation, and comparison of clinical samples and controls.

Amplicon sorter was then used to group reads from samples based on their gene associations. Samples meeting the following criteria were then used to generate consensus fasta files: 850–1500 bp length read and >1000 reads. Consensus fasta files were then classified using the rRNA/ITS database via BLAST. Results with a minimum of >95% percentage identity and >90% coverage in comparison to a database entry were then taken forward for identification according to The CLSI guideline MM-18 A (Vol. 28 No 12): Interpretive Criteria for Identification of Bacteria and Fungi by DNA Target Sequencing.^17^

### Determining the clinical demand and impact of introducing ONT diagnostics

Data was collected from 7 different hospitals including a paediatric and a cancer hospital from the introduction of the test on 01/05/23 to 15/02/24. A total of 218 samples were collected from 188 patients (see Supplementary Files 1 and 2).

For each case, the following data were obtained from the laboratory database and patient notes: source and result of sample, patient age, patient sex, patient location, immune status, date of admission and date of discharge. Antibiotic appropriateness was assessed in reference to local trust guidelines, aligned with national and NICE standards. Broad-spectrum antibiotics are initially prescribed; 16S results were then used to tailor therapy, switching to narrow-spectrum agents, enabling oral therapy, or stopping antibiotics entirely. Clinical notes were reviewed to evaluate whether therapy was changed, stopped or unchanged (no change).

The primary outcome of interest was the clinical decision following the test, specifically, whether the 16S result prompted a modification in antimicrobial therapy and where these modifications had the greatest clinical impact (defined as the subpopulation exhibiting the highest odds ratio (OR) for a management change prompted by the 16S result). To assess this, we classified clinical metadata related to antimicrobial changes into three categories: escalation (change of antibiotic therapy or initiation of antibiotics), rationalisation (confirms non-infectious cause, confirms treatment choice, stopping antibiotics or stopping antibiotics and changing treatment), and unclear (all other cases).

### Statistics

We used logistic regression to evaluate:

1) Whether there is an association between the clinical impact of 16S rRNA sequencing, and immunocompromised status (immunocompromised or immunosuppressed). Given the heightened vulnerability of individuals who are immunocompromised to infections^18^ and the clinical importance of timely, targeted antimicrobial therapy in this group,^19^ assessing the diagnostic utility of 16S sequencing in this context is critical.
2) Whether the clinical impact of 16S differs between males and females. Existing literature reports a higher burden of antimicrobial resistance (AMR) genes in patients who are recorded as female.^20^ As such, it is important to understand whether molecular diagnostics yield differential benefits or lead to divergent clinical decisions based on sex.
3) Whether the clinical impact of 16S differs based on the age of the patient. Older adults represent a uniquely vulnerable population due to immunosenescence, multimorbidity, polypharmacy, and structural disparities in healthcare access.^21^ These factors, along with atypical presentations of infection, often result in diagnostic delays and poorer clinical outcomes.^22^ Evaluating the age-specific utility of 16S sequencing is therefore essential to guide diagnostic stewardship in an ageing population.

Clinical impact was assessed using two complementary outcome definitions; a binary actionable outcome, which was defined as any change in antimicrobial management (escalation or rationalisation; assessed using the R package stats v4.4.2), and a multinomial outcome in which escalation, rationalisation and unclear were assessed independently (assessed using the R package nnet v7.3.20). All analyses were performed at the patient level to maintain the independence of observations. See Supplementary Fig. S1 for a summary.

### Role of funders

Liverpool Clinical Laboratories (LCL) funded this work, and played a role in the study design, data collection and writing of this report.

## Results

### Assay validation

#### 16S sequencing using ONT improves results for accreditation from the previous clinical pathway, and accurately characterises polymicrobial clinical samples

All 109 samples produced results in line with those issued by QCMD or identified from our own samples previously, (overall consistency = 100%), including an additional educational sample provided by QCMD that had two different organisms present (*Klebsiella pneumoniae* & *Acinetobacter baumannii*). This sample, when originally tested and reported in 2021 via the previous method, was reported as ‘unable to sequence’ due to the mixed composition of the sample. When tested as part of this exercise however, the two different organisms were both correctly identified. QCMD results are summarised in Table 2.

**Table 2:** Comparison of taxonomy expected for accreditation and ONT results (QCMD samples only).

QCMD ID	ONT ID
*K. pneumoniae* & *A. baumannii*	*K. pneumoniae* & *A. baumannii*
*Microbacterium maritypicum*	*Microbacterium maritypicum*
*Escherichia coli*	*Shigella*/*Escherichia* sp.
*Staphylococcus epidermidis*	*Staphylococcus epidermidis*
*Yersinia enterocolitica*	*Yersinia enterocolitica*

The ONT protocol produced organism matches or improved diagnosis in all clinical samples included in assay validation. For the 8 samples where the previous Sanger sequencing method failed to produce a sequence, culture and MALDI-TOF were used to obtain an organism for comparison, with all 8 identifications matching. Among the 24 samples reported as negative by the reference laboratory, the ONT assay results were consistent for 21 samples, but bacterial DNA was amplified for 3 samples, including a *Fusobacterium* sp. in a CSF sample and *Pseudomonas aeruginosa* in two fluid samples.

In samples referred to the reference laboratory, targeted PCR was used instead of the 16S sanger sequencing. In these cases, ONT could be used to identify the reported organisms on the basis of 16S alone, and identified three additional organisms (Table 3).

**Table 3:** Comparison of reference laboratory and ONT results.

Reference laboratory results	ONT results
*Kingella kingae*	*Kingella kingae, Pseudomonas aeruginosa*
*Kingella kingae*	*Kingella kingae, Sphingomonas* sp., *Acinetobacter* sp.
*Neisseria meningitidis*	*Neisseria meningitidis*
*Streptococcus pyogenes*	*Streptococcus pyogenes*
*Streptococcus pyogenes*	*Streptococcus pyogenes*

### Patient and sample demographics and assay clinical impact

#### The largest demographic for sample requests were inpatients and patients aged 50-69

**Fig. 1:**
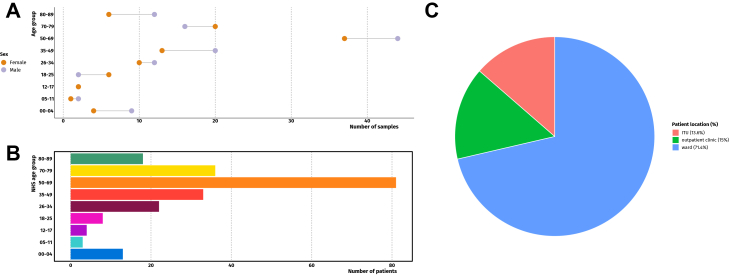
**Patient demographics and sample origin.** (A) Number of samples against the age and sex distribution of patients. (B) The number of samples taken across all age groups. (C) Sample origin by patient location. Patients originated from 7 different trusts including acute trusts and specialist neurological, surgical, oncology and paediatric centres. Most outpatient requests originated from Ophthalmology. 13.0% of samples originated from ITUs. For comparison the largest acute trust (LUHFT) included in the data collection has ∼5.5% critical care beds. The sampling had a bias towards critical care admissions, reflecting sicker patients with longer stays.

Patients originated from 7 different trusts, including acute trusts and specialist neurological, surgical, oncology, and paediatric centres (218 samples across 188 patients; see Fig. 1). Of these patients, 107 were male (56.9%); and 81 were female (43.1%). Additionally, 141 patients (75.0%) were immunocompetent, 22 (11.7%) were immunosuppressed, whilst no information on immune competence was available for 25 individuals (13.3%). In terms of age demographics, the median patient age was 59 (IQR: 34.5–69). The highest representation was in the 50–69 age group (81 patients), followed by 70–79 (36 patients). This reflects the increased burden of infection and diagnostic uncertainty in older adults. The smallest demographic sampled was the 05–11 age group (3 patients). Across six out of nine age brackets, more patients were recorded as male than female.

The majority (71.9%) of samples were from patients on wards, followed by outpatient clinics (15.0%) and intensive care units (ITUs; 13.0%) (see Fig. 1). The majority of outpatient requests originated from Ophthalmology. The largest acute trust (LUHFT) included for data collection has ∼5.5% critical care beds.

#### 16S sequencing effectively addresses culture-negative cases, confirms or optimises antimicrobial therapy

The main reason for requesting 16S sequencing was the inability to culture organisms, representing 32.1% of cases. A similar proportion (32.6%) involved samples taken while patients were on antibiotics. Other reasons included confirming a non-infectious cause (5%), suspicion of fastidious organisms (4.1%), and concerns over turnaround time (3.7%). Smaller percentages related to difficulties identifying organisms (1.8%) or other unspecified reasons (9.2%), with 11.5% of cases lacking a recorded rationale.

The most frequent outcome following 16S sequencing was no change in therapy (28%), followed by confirmation of the current treatment choice, occurring in 26.6% of cases. Notably, 16S sequencing influenced antimicrobial stewardship by prompting a change in antibiotic therapy in 8.3% of cases and stopping antibiotics in 16.5%. Less common clinical impacts included confirming a non-infectious cause (3.7%), initiating antibiotic therapy (1.4%), and stopping antibiotics while starting alternative treatment (0.5%). In 15.1% of cases, the clinical impact was undocumented. These findings highlight the utility of 16S sequencing in improving diagnostic certainty, thereby supporting more precise, evidence-based antimicrobial decision-making, including decisions to not modify therapy.

**Fig. 2:**
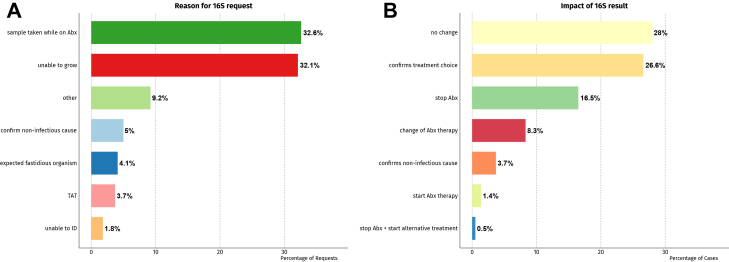
**Requests for 16S analysis support antimicrobial stewardship efforts and are most common when bacterial culture is unsuccessful.** (A) Summary of the 16S requests. The majority of requests originated from failure to grow with conventional culture methods. (B) Summary of clinical decision-making post 16S result. Most outcomes either confirmed treatment choice (26.6%) or resulted in no change (28%), however the test contributed to antimicrobial stewardship in 56.2% of cases through either escalation (8.3%) or rationalisation (48.6%) of antibiotic regimen, including stopping or narrowing of therapy.

A summary of reasons for requesting 16S sequencing and clinical outcomes are shown in Fig. 2.

#### Streptococcus and Staphylococcus are found in most samples, with Streptococcus more linked to hospitalisation

*Streptococcus* was the most frequently identified genus, accounting for 33.3% of detections in the intensive care unit (ITU) and 15.4% on inpatient wards. *Staphylococcus* was only found on the ward (7.7%) and was identified in cerebrospinal fluid (CSF) samples, other fluid samples and tissue samples. *Cutibacterium* showed notable prevalence in ITU-based samples (16.7%) and was frequently associated with tissue (16.7%), pus or abscess material (21.4%), consistent with its role in skin and soft tissue infections, but was also identified in joint fluid (25.0%). *Fusobacterium* was detected across all patient locations and was identified in pus or abscess (21.4%) and fluid samples (12.5%), whereas *Mycobacterium* and *Parvimonas* were only found in outpatient samples (Tissue and Swab samples respectively). *Streptococcus* remained the most detected genus in CSF (28.6%) and fluid specimens (37.5%), and was also identified in blood (20.0%), swab (33.3%), and pus samples (14.3%).

**Fig. 3:**
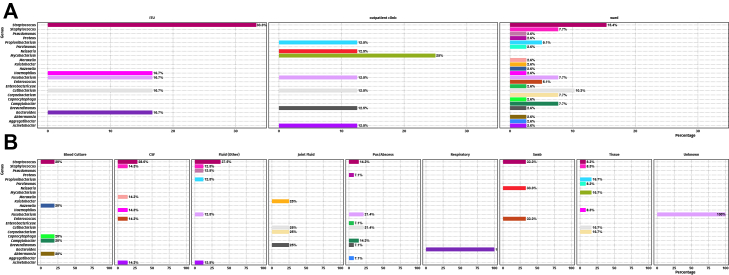
**Genus-level distribution of bacterial taxa detected by ONT 16S sequencing.** (A) Distribution by patient location (ITU, outpatient, ward). (B) Distribution by sample type. Percentages reflect the proportion of each genus within the category.

A summary of all organisms identified across the samples in this study can be found in Fig. 3 and Supplementary File S3.

#### 16S sequencing has the greatest antimicrobial stewardship impact in patients who are immunosuppressed; age and sex are not significantly associated with escalation or rationalisation

Categorisation of 16S results into those that contributed to antimicrobial stewardship (escalation or rationalisation) and those that did not showed that 124/218 (56.9%) of 16S samples effectively contributed to antimicrobial stewardship (21 = escalation, 103 = rationalisation – see Table 4 and Supplementary File S2). 16S was most useful for patients who are immunosuppressed, who had significantly higher odds of antimicrobial stewardship impact (OR = 2.99, 95% CI [1.11–9.50], p = 0.04 [Wald test, logistic regression]) compared to patients who are immunocompetent. In contrast, there was no significant association between sex (OR = 0.85, 95% CI [0.47–1.54], p = 0.60 [Wald test, logistic regression]) or age (OR = 1.00, 95% CI [0.98–1.01], p = 0.68 [Wald test, logistic regression]) and antimicrobial stewardship (see Table 5). Multinomial analysis considering escalation and rationalisation as separate outcomes yielded similar results. Individuals who are immunocompromised had significantly higher odds of escalation in comparison to individuals who are immunocompetent. The odds for rationalisation were elevated, but not significant. Age was not significantly associated with the odds of escalation (OR = 1.03, 95% CI [1.00–1.06], p = 0.08 [Wald test, logistic regression]) or rationalisation (OR = 1.75, 95% CI [0.84–3.65], p = 0.14 [Wald test, logistic regression]). Sex was also not significantly associated with the odds of escalation (OR = 1.08, 95% CI [0.38–3.10], p = 0.88 [Wald test, logistic regression]) or rationalisation (OR = 0.82, 95% CI [0.44–1.51 [Wald test, logistic regression]], p = 0.52). Despite 16S being most useful for patients who are immunosuppressed, wide confidence intervals indicate limited precision in this estimate, reflecting the available sample size. However, the direction of effect remains consistent, and across all patient groups, 16S sequencing contributed clinical value by reducing diagnostic uncertainty, which supports more targeted antimicrobial use.

**Table 4:** Clinical impact of 16S results on antimicrobial stewardship: escalation, rationalisation, or no effect.

Clinical impact category	n	%
Rationalisation	**103**	**47.25**
Confirms non-infectious cause	8	
Confirms treatment choice	58	
Stop antibiotics	36	
Stop antibiotics + start alternative treatment	1	
Escalation	**21**	**9.63**
Change of antibiotic therapy	18	
Start antibiotic therapy	3	
Unclear	**94**	**43.12**
No change	61	
NA	33

**Table 5:** Association between the clinical impact of antimicrobial stewardship and patient age, sex and immune status (logistic regression).

Variable	p-value	95% CI	Odds Ratio	Reference level
Age	0.68	0.98–1.01	1.00	None
Immune status	0.04	1.11–9.50	2.99	Competent
Sex	0.60	0.47–1.54	0.85	Female

## Discussion

In this study, we have successfully implemented 16S sequencing using Oxford Nanopore Technology (ONT) as part of an NHS-accredited assay for rapid bacterial diagnosis and identification. This method offers a turnaround time (TAT) of 24–72 h, which is faster than traditional methods (e.g., 16S PCR from culture, or MALDI-TOF for bacterial identification), or the previous method that took approximately seven days from sample receipt. With complete on-call coverage and optimal staffing levels, TAT would consistently be around 24 h. Critically we have been able to show that the assay results have influenced clinical decision-making, impacting antimicrobial stewardship in 56.2% of cases, with the greatest clinical impact in patients who are immunocompromised. Similar approaches have since been developed in other NHS trusts,^23^ highlighting the growing recognition of this technology’s potential to transform infectious disease diagnostics.

ONT offers several advantages over traditional Sanger sequencing, allowing the sequencing of the entire 16S gene in a single contig and eliminating the need for sequence reassembly.^24^ Here, we have shown that we were able to enhance service delivery without sacrificing reliability, which is a common concern for culture-independent assays.^25^ The only discrepancies reported during assay validation and verification were cases where ONT detected additional organisms compared to previous methods or provided species-level identification where the older methods could only offer genus-level classification. The enhanced sensitivity during method validation resulted in four instances where the ONT method detected additional bacterial organisms compared to the previous method and three cases where ONT identified bacteria in samples that the reference laboratory had reported as negative. These findings had the potential to pose challenges for clinicians, as the presence of multiple microorganisms could complicate interpretation,^26^ which is often the case with metagenomic approaches.^27^ However, the clinical review process for 16S rRNA sequencing minimised potential misinterpretations, such as the inappropriate treatment of detected colonisers. This was achieved by ensuring all results were meticulously reviewed by a team of microbiology consultants who correlated genomic data with clinical scenarios and standard culture results. To support this interpretation, extensive training was provided to clinical teams to familiarise them with the sequencing methods and the bioinformatic pipelines used to identify pathogens. Methodologically, potential errors were further reduced through strict pre-analytic controls and the systematic use of negative and extraction controls to monitor for reagent contamination.

With the launch of the 16S ONT assay, we have successfully demonstrated the feasibility of integrating clinical metagenomics into routine diagnostics. Metagenomic approaches tend to be novel methods that are routinely confined to very complex cases with usage guidelines determined by lack of accessibility, prohibitive costs and long turnaround times.^8^ Our aim was to develop an accessible metataxonomic assay, free of these constraints, while collecting data on the usage of the assay as well as on the subsequent impact on patient care. This was with a view of introducing evidence-based guidelines, optimising the use of these highly effective tools.

To this end, we have collected data both of sample and patient demographics throughout the first 9 months since the assay launch, as well as on the clinical impact as determined by the ward teams of the results of the 16S assay. Since its introduction, the assay has been adopted by seven hospitals across Cheshire and Merseyside. Samples from all sterile sites were accepted for 16S with “pus”, “fluid” and “tissue” accounting for the majority of these. These reflect infectious conditions that would both be challenging for antibiotic penetration (such as abscesses), as well as potentially requiring prolonged antibiotic treatment (such as in joint fluids and biopsies, or infected grafts).^28^

Unsurprisingly, the primary reason for 16S testing was the failure of conventional culture methods to identify the organism, or a reduction of the likelihood that the culture would be successful, due to previous therapy. This points to a systematic delay in the request of the 16S assay, although with an inevitable sampling bias, as the assay would be requested if routine methods failed to produce a result or an antibiotic regimen was deemed to be ineffective. This bias also means that we cannot assess the clinical impact of this assay on an unselected patient population.

A key limitation of 16S rRNA sequencing is the absence of antimicrobial susceptibility information. Whilst our method provides rapid identification, it cannot detect antimicrobial resistance genes or predict susceptibility profiles. In principle, this can be achieved using genomics (whole-genome sequencing or shotgun metagenomics). However, for most bacteria, the relationship between genotype and phenotype remains incompletely validated. As such, culture is still essential for reliable diagnosis and treatment. Future work will aim to establish metagenomic assays using a similar framework, which have the benefit of characterising polymicrobial infections at higher resolution in a cultivation-independent manner,^29^ whilst simultaneously enabling the identification of AMR genes for additional confirmation of risk in the identified pathogen (e.g., on plasmids; which cannot be achieved with 16S rRNA sequencing alone), as well as identification of at-risk individuals.^30^

Whilst any new assay that impacts antibiotic treatment must be used carefully, antibiotic de-escalation can be safely guided by 16S rRNA identification alone, using a multimodal framework that integrates microbiological rules and guidelines, epidemiological data, and clinical safety indicators. ID-only guided de-escalation relies on applying “expected phenotypes” and intrinsic resistance tables from expert bodies such as EUCAST, noting that certain pathogens, such as *Streptococcus pyogenes,* remain universally susceptible to penicillin.^31^ Localised antibiograms to select targeted agents with a high probability of coverage can also be used.^32^ Large-scale clinical evidence confirms that de-escalating broad-spectrum therapy by day 4 in patients who are clinically stable does not compromise survival and significantly reduces both antibiotic exposure and hospital length of stay.^33^ Furthermore, the high negative predictive value of 16S rRNA sequencing in sterile-site samples allows for safe antibiotic discontinuation when no bacterial DNA is detected, provided clinicians continue to monitor for alternative diagnoses as recommended by international guidelines.^34^

We also recognise that the observed associations between clinical decisions based on 16S results with factors such as age, sex, and immune status may be affected by confounding factors such as medication^35^ or pathogen abundance.^36^ Since this was a descriptive implementation study without a control group, it is not possible to establish causality. Additionally, owing to the limited availability of clinical material, where associations were observed, confidence intervals were also wide (see immune status in Table 5). Finally, clustering by hospital was not explicitly modelled. As such, estimates may be subject to residual clustering, which could affect the precision of confidence intervals. However, the number of contributing hospitals was small (n = 7), and the primary aim was to estimate overall associations rather than hospital-specific effects. Despite this, the observed associations were consistent with clinically meaningful impacts across different hospitals.

In conclusion, it is recognised that rapid and effective diagnostics are the first step towards improved antimicrobial stewardship leading to both a reduction and an optimisation of the use of antibiotics. To this end we have shown that the optimised ONT 16S workflow offers a sensitive, specific, and rapid method for identifying bacterial organisms. The workflow successfully addresses the limitations of the traditional Sanger method and has become an invaluable component of our routine diagnostic repertoire. Future applications of this assay are being explored, including further optimisation for different sample types, such as microbial keratitis and water contamination monitoring at local hospitals. We have additionally demonstrated that more research is required to identify samples where routine culture would be less successful.

This would involve considering both patient- and sample-level characteristics, including samples with a high probability of fastidious organisms such as mycobacteria, and samples from patients with a history of antimicrobial therapy.

## Contributors

We describe author contributions to the paper using the CRedit taxonomy. Conceptualisation: A.S. and A.C.D. Data curation: A.S., E.C.-O., C.E., S.L., A.L., C.L., M.S., N.S., L.S. and V.O. Formal analysis: E.C.-O., D.C., J.B., M.M., A.R. and J.P. Funding acquisition: A.S. Investigation: E.C.-O., A.S. and A.C.D. Methodology: E.C.-O., A.S. and A.C.D. Project administration: A.S. Resources: A.S., A.C.D. and A.F. Software: A.S. and A.F. Supervision: A.S. and A.C.D. Validation: E.C.-O. and A.S. Visualisation: E.C.-O. Writing – original draft: E.C.-O. Writing – review and editing: all authors. All authors read and approved the final version of the manuscript. A.S. and E.C.-O have accessed and verified the underlying data.

## Data sharing statement

The clinical metadata and analysis code supporting the findings of this study are publicly available. De-identified clinical metadata (CSV format), along with the full analysis pipeline (R scripts) and code to reproduce all figures and tables, are accessible via GitHub at: https://github.com/edwardcunningham-oakes/16S-stewardship-impact.

These materials are available upon publication of this manuscript and will remain accessible without a specified end date.

## Declaration of interests

E.C.-O. was Chair of EDI (Members Panel) for the Microbiology Society during 2022–2024. This was a voluntary role and outside the scope of the submitted work. D. C., J. B., A. F., M. M., C. E., S. L., A. L., C. L., M. S., N. S., L. S., V. O., and A. S. are NHS employees.

## Appendix A Supplementary data

Supplementary File S3

Supplementary Fig. S1 Supplementary Fig. S1: Graphical summary of our workflow from patient sample to statistical analysis.
