# Supplementary figures and images for: Implementing portable, real-time 16S rRNA sequencing in the healthcare sector enhances antimicrobial stewardship
Source: eBioMedicine. 2026 Jun 10;129:106317. doi: 10.1016/j.ebiom.2026.106317 (PMC13347615; doi:10.1016/j.ebiom.2026.106317)

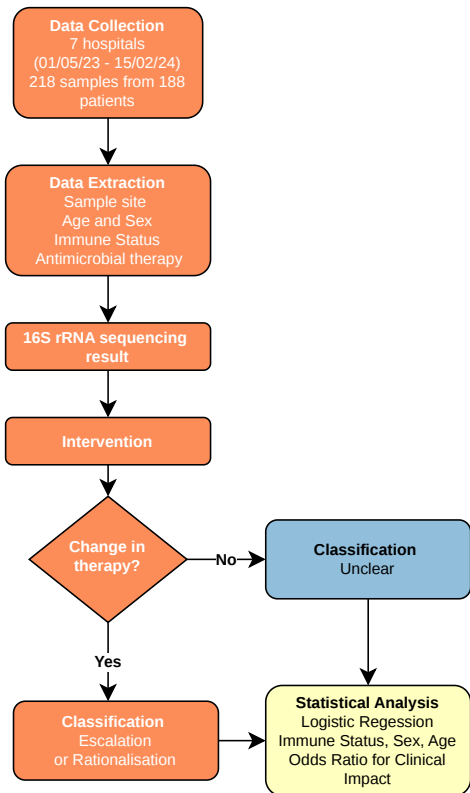

Supplement: Supplementary Fig. S1 — Graphical summary of our workflow from patient sample to statistical analysis. [file mmc4.pdf]
